# Crystal structures of HER3 extracellular domain 4 in complex with the designed ankyrin-repeat protein D5

**DOI:** 10.1107/S2053230X21006002

**Published:** 2021-06-29

**Authors:** Filip Radom, Clemens Vonrhein, Peer R. E. Mittl, Andreas Plückthun

**Affiliations:** a Department of Biochemistry, University of Zurich, Winterthurerstrasse 190, CH-8057 Zürich, Switzerland; b Global Phasing Ltd, Sheraton House, Castle Park, Cambridge CB3 0AX, United Kingdom

**Keywords:** protein engineering, DARPins, HER3, EGFR family, tumor targeting, protein engineering

## Abstract

The members of the human epidermal growth factor receptor (HER) family are among the most intensely studied oncological targets. HER3 (ErbB3), which had long been neglected, has emerged as a key oncogene, regulating the activity of other receptors and being involved in progression and tumor escape in multiple types of cancer. Designed ankyrin-repeat proteins (DARPins) serve as antibody mimetics that have proven to be useful in the clinic, in diagnostics and in research. DARPins have previously been selected against EGFR (HER1), HER2 and HER4. In particular, their combination into bivalent binders that separate or lock receptors in their inactive conformation has proved to be a promising strategy for the design of potent anticancer therapeutics. Here, the selection of DARPins targeting extracellular domain 4 of HER3 (HER3d4) is described. One of the selected DARPins, D5, in complex with HER3d4 crystallized in two closely related crystal forms that diffracted to 2.3 and 2.0 Å resolution, respectively. The DARPin D5 epitope comprises HER3d4 residues 568–577. These residues also contribute to interactions within the tethered (inactive) and extended (active) conformations of the extracellular domain of HER3.

## Abstract 2

The structure of a designed ankyrin-repeat protein that selectively binds to domain 4 of HER3, an important driver of malignant growth in many tumors, has been determined. The structure helps to explain the selectivity against other members of the HER family, and binding to this epitope will interfere with its interactions in the tethered (inactive) and extended (active) conformations.

## 1. Introduction   

The human members of the epidermal growth factor receptor (HER, ErbB) family are membrane receptors that are involved in cell division, survival and migration (Yarden & Sliwkowski, 2001 ▸). HER proteins comprise four extracellular domains, a transmembrane domain, an intracellular kinase domain and a long unstructured C-terminal tail that carries phosphorylation sites which can be bound by adaptor proteins. Homodimerization and heterodimerization, usually induced by ligand binding, promote mutual phosphorylation of the kinase domain and the C-terminal tail, which in turn activates a variety of signaling cascades (Hynes & MacDonald, 2009 ▸). The extracellular domain of HER3 (UniProt entry P21860) is subdivided into four domains, namely domain 1 (residues 1–183; numbering of the mature protein, not counting the 19 residues of the signal sequence), domain 2 (residues 184–308), domain 3 (residues 309–480) and domain 4 (residues 481–611).

In the absence of its ligand, HER3 exists predominantly in a tethered conformation in which extracellular domains 2 and 4 form a contact (Cho & Leahy, 2002 ▸). Upon binding its natural ligand, such as neuregulin-1 or neuregulin-2 (also known as heregulins), via extracellular domains 1 and 3, the extracellular domains are structurally rearranged into an upright form. Extracellular domain 2 can then promote dimerization with other receptors from the HER family. Since the kinase domain of HER3, unlike other kinase domains from the HER family, displays only minimal activity, HER3 becomes signaling-active exclusively upon heterodimerization (Jura *et al.*, 2009 ▸), since only then does the C-terminal tail become phosphorylated and induce further cell-signaling events. The main downstream signaling proteins of HER3 are protein kinase B (AKT) and mitogen-activated protein kinases (MAPKs), which both trigger different cell-proliferation mechanisms (Yarden & Sliwkowski, 2001 ▸).

Overexpression of HER3 is associated with the development of multiple cancers, including breast, lung, prostate, gastric, bladder, melanoma, colorectal and squamous cell carcinomas (Beji *et al.*, 2012 ▸; Hayashi *et al.*, 2008 ▸; Lipton *et al.*, 2013 ▸; Luckow *et al.*, 1993 ▸; Nielsen *et al.*, 2015 ▸; Qian *et al.*, 2015 ▸; Reschke *et al.*, 2008 ▸; Tanner *et al.*, 2006 ▸; Siegfried *et al.*, 2015 ▸). In most cases, tumor growth is coupled to the overexpression of other HER family members, which serve as the preferred HER3 heterodimerization partners (Liles *et al.*, 2010 ▸; Travis *et al.*, 1996 ▸). For example, the active HER3–HER2 heterodimer has emerged as an important oncogenic unit in breast cancer, while the HER3–HER1 heterodimer drives melanoma and pancreatic carcinoma (Reschke *et al.*, 2008 ▸; Liles *et al.*, 2010 ▸). Thus, preventing HER heterodimerization is an important strategy for preventing malignancy (Mishra *et al.*, 2018 ▸).

Designed ankyrin-repeat proteins (DARPins) constitute a class of artificial repeat proteins that were developed as alternatives to antibodies. They consist of N- and C-terminal capping repeats and typically 2–3 internal repeats. The 33-amino-acid internal repeats are randomized in DARPin libraries at positions 2, 3, 5, 13, 14 and 33 (Binz *et al.*, 2004 ▸). Selection methods, such as ribosome or phage display, allow the enrichment of DARPins with specific residues at the randomized positions that confer selectivity for a particular target. Specific DARPins targeting HER1 (EGFR), HER2 and HER4 have been selected (Steiner *et al.*, 2008 ▸; Zahnd *et al.*, 2006 ▸). Knowledge of the epitopes in different domains of the receptor was crucial for the subsequent design of bispecific constructs. For example, DARPin E01, when connected by a leucine zipper to DARPin E69, reduces cell proliferation by inhibiting EGFR recycling (Boersma *et al.*, 2011 ▸). DARPin G3 flexibly linked to DARPin 9_26 can prevent HER2 homodimerization and induces apoptosis more strongly than any approved antibody-based drug (Jost *et al.*, 2013 ▸; Tamaskovic *et al.*, 2016 ▸). These examples show the great potential of HER-directed DARPins for the development of anticancer drugs.

In this study, we report the selection and structural characterization of DARPins binding to HER3 extracellular domain 4 (HER3d4), because these DARPins could be used to generate bispecific constructs with diverse functions, for example molecules that are able to lock the receptor into an inactive conformation, in analogy to the strategy reported previously (Jost *et al.*, 2013 ▸).

## 2. Materials and methods   

### 2.1. Expression and purification of HER3 domains for DARPin selection   

HER3 domain 4 (residues 500–643 of UniProt entry P21860) with an N-terminal AviTag and His_6_ tag for selection and purification was cloned into a pFL shuttle vector for subsequent expression in insect cells (Table 1 ▸; Trowitzsch *et al.*, 2010 ▸; Fitzgerald *et al.*, 2006 ▸). Transformation of the *Escherichia coli* EmBacY strain, isolation of the baculoviral genome, transfection of *Spodoptera frugiperda* (Sf9) cells and amplification of the virus were performed according to established protocols (Murhammer, 2007 ▸). Sf9 cells were cultured in SF900II medium (Thermo Fisher Scientific). For expression, 4 × 10^5^ cells ml^−1^ were infected with virus at a multiplicity of infection of 5 and incubated in suspension for 96 h at 27°C with orbital shaking (90 rev min^−1^, 25 mm rotor radius). The cells were harvested by centrifugation (7000*g*, 20 min, 4°C) and the supernatant containing the secreted protein was subjected to immobilized metal ion-affinity chromatography (IMAC) purification with Ni-Superflow purification resin (2 ml beads per 1 l culture). *In vitro* biotinylation was performed according to a protocol from Avidity. Biotinylated HER3d4 for DARPin selection was purified by size-exclusion chromatography using a Superdex 75 10/300 GL column (GE Healthcare). HER2 domain 4 (HER2d4; residues 539–625), used for counter-selection against binders cross-reactive to HER2, was expressed and purified using the same procedure.

### 2.2. Selection and characterization of DARPins   

DARPins binding to HER3d4 were selected by ribosome display with the biotinylated target immobilized on a plate as described previously (Zahnd *et al.*, 2007 ▸; Hansen *et al.*, 2017 ▸). Selection conditions are summarized in Supplementary Table S1. To favor specific and high-affinity binders, selection included additional pre-panning steps against HER2d4, random mutagenesis and incubation with excess non-biotin­ylated HER3d4 competitor for off-rate selection. Error-prone PCR with nucleotide analogs was introduced after panning round 3 to generate approximately 1–2 mutations per DARPin sequence (Zaccolo *et al.*, 1996 ▸).

DNA of selected binder pools was cloned into the pQiq_FLAG expression vector, containing an N-terminal His_6_ tag and a C-terminal FLAG tag flanking the DARPins, and *E. coli* XL1 Blue cells (Stratagene) were transformed. Single clones were picked to start expression in a 100 µl volume of Terrific Broth (TB) medium in a 96-well plate (Greiner). Expression was induced with 0.5 m*M* isopropyl β-d-1-thiogalactopyranoside (IPTG) and was allowed to continue for 3 h at 37°C with orbital shaking (250 rev min^−1^, 50 mm rotor radius). The harvested cells were lysed with B-PER cell-lysis buffer (Thermo Scientific) and diluted 1000-fold in PBS-B (phosphate-buffered saline with 0.2% BSA). Diluted crude extracts were incubated with 8 n*M* biotinylated target. For detection by homogeneous time-resolved fluorescence (HTRF), terbium cryptate-conjugated streptavidin (Streptavidin-Tb; Cisbio, Part No. 610SATLB) was used as a FRET donor and anti-FLAG (M2) antibody conjugated with the dye d2 (Cisbio, Part No. 61FG2DLB) was used as a FRET acceptor. A CyBi-FeliX (Cisbio) robot system was used for pipetting and measurements.

For analytical size-exclusion chromatography and further characterization, selected binders were expressed on a larger scale and purified. Briefly, single clones were grown in 1 ml TB medium in a 96-deep-well plate (Greiner). Expression was induced with 0.5 m*M* IPTG and continued for 6 h at 37°C with orbital shaking (550 rev min^−1^, 12.5 mm rotor radius). The harvested cells were lysed with CelLytic B (Sigma) in equilibration buffer (50 m*M* sodium phosphate, 0.3 *M* NaCl, 50 m*M* MgCl_2_, 80 U ml^−1^ Pierce nuclease from Thermo Scientific). Lysates were purified with HisPur Cobalt Spin Plates (Thermo Scientific) according to the manufacturer’s protocols. Analytical size-exclusion chromatography was performed on an ÄKTAmicro system with a Superdex 200 Increase 5/150 GL column (GE Healthcare).

Affinities were measured by surface plasmon resonance on a ProteOn XPR36 instrument equipped with a NeutrAvidin-containing NLC chip (Bio-Rad) in PBS supplemented with 0.005% Tween-20. Two ligand channels were coated with biotinylated HER3d4. Monomeric DARPins were injected at flow rates of 60 µl min^−1^ at five increasing concentrations for 5 min (duplicate measurements at 2.5, 7.5, 22, 67 and 200 n*M*), followed by a dissociation phase of 5 min. Data were double-referenced and fitted to a kinetic titration model using the *ProteOn Manager* and *BiaEvaluation* software (Karlsson *et al.*, 2006 ▸).

### 2.3. Macromolecule production   

HER3d4 without an AviTag was expressed and purified from insect cells as described above. The protein was enzymatically deglycosylated by incubating glycosylated HER3d4 with PNGase F (NEB; 5 µl PNGase F per 1 mg HER3d4) in a dialysis bag (3 kDa molecular-weight cutoff) against PBS overnight at 4°C. The remaining glycosylated HER3d4 was removed with ConA Sepharose 4B beads (GE Healthcare). The molecular mass was confirmed by mass spectrometry.

DARPins with an N-terminal His_6_ tag followed by a 3C protease site were expressed in *E. coli* XL1 Blue in 400 ml TB medium for 5 h at 37°C with orbital shaking (90 rev min^−1^, 50 mm rotor radius). Expression was induced with 0.5 m*M* IPTG. The cells were sonicated and the supernatant was filtered and purified with Ni–NTA Sepharose as described previously (Binz *et al.*, 2003 ▸). The His_6_ tag was removed by 3C protease cleavage at a molar ratio of 1:100 (protease:DARPin) and dialyzed against PBS. The solution was applied to equilibrated Ni–NTA beads and incubated for 1 h at 4°C with constant rotation. The beads were filtered and the flowthrough containing the DARPin lacking the His_6_ tag was recovered. The correct masses of the proteins were confirmed by mass spectrometry. DARPin D5 was incubated with HER3d4 for 0.5 h at a 1:1.2 molar ratio and the complex was purified by gel filtration (S-200 column, GE Healthcare, equilibrated with PBS). Finally, the complex was concentrated to 7.2 mg ml^−1^ with a Millipore Amicon Ultra 3K centrifugal concentrator.

### 2.4. Crystallization   

A Phoenix crystallization robot (Art Robbins Instruments) was used to set up sitting-drop vapor-diffusion experiments in 96-well plates. Initial crystallization conditions were identified by sparse-matrix screens from Hampton Research (California, USA) and Molecular Dimensions (Suffolk, England) and were subsequently refined by grid screens in which the pH and the PEG concentrations were varied simultaneously. Protein solutions were mixed with reservoir solutions in 1:1, 1:2 and 2:1 volume ratios (200 or 300 nl final volumes), and the mixtures were equilibrated against 75 µl reservoir solution at 4°C. Reservoir conditions are specified in Table 2 ▸. Spherulites of HER3d4–DARPin D5 formed under reservoir conditions containing 200 m*M* salt (either lithium or ammonium sulfate), with a pH of between 4 and 6, and containing medium PEG concentrations of between 25% and 30%. Crystalline particles from similar conditions were collected and used for two rounds of micro-seeding (Seed Beads, Hampton Research). Crystals grew within five days under very similar conditions (Table 2 ▸).

### 2.5. Data collection, structure solution and refinement   

Crystals were transferred to reservoir solution supplemented with 25%(*v*/*v*) ethylene glycol as a cryoprotectant, mounted in cryo-loops from Hampton Research and flash-cooled in liquid nitrogen. Data were collected on beamline X06SA at the Swiss Light Source (Paul Scherrer Institute, Villigen, Switzerland) and were processed with *XDS* and *autoPROC* (Table 3 ▸; Kabsch, 2010 ▸; Vonrhein *et al.*, 2011 ▸). Due to inappropriate centering of the crystal, some frames were subsequently excluded from processing. For the orthorhombic crystal we scaled frames 1–650, 1200–2300 and 3100–3600, and for the monoclinic crystals we used frames 851–1950 and 2551–3600. Structures were determined by molecular replacement using *MOLREP* (Vagin & Teplyakov, 2010 ▸). We first determined the structure of the monoclinic crystal form using the structures of DARP-3.4 (PDB entry 2y0b; Schroeder *et al.*, 2013 ▸) and HER3d4 (PDB entry 4leo; Mirschberger *et al.*, 2013 ▸) as search models. After initial refinement of the complex in the monoclinic crystal form, we used the preliminary HER3d4–DARPin D5 complex to determine the structure in the orthorhombic crystal form. Structures were refined using *REFMAC*5 and *BUSTER* (Murshudov *et al.*, 2011 ▸; Bricogne *et al.*, 2017 ▸). Refinement statistics are given in Table 4 ▸. For model building and preparation of figures, we used *Coot* and *PyMOL* (Emsley *et al.*, 2010 ▸; DeLano, 2002 ▸). Structures were analyzed using the *PISA* server and *SC* (Krissinel & Henrick, 2007 ▸; Lawrence & Colman, 1993 ▸). The structures were deposited in the PDB with accession codes 7bhe (monoclinic crystals) and 7bhf (ortho­rhombic crystals), and raw diffraction data were deposited at https://www.proteindiffraction.org/.

## 3. Results   

### 3.1. Selected DARPins against HER3d4   

We selected DARPins against HER3d4 by ribosome display using established procedures (Dreier & Plückthun, 2012 ▸). The selection was performed using the N2C and N3C libraries with scaffolds comprising two and three internal repeats, respectively. The selection yielded eight N2C and ten N3C hits in time-resolved fluorescence (HTRF) binding analysis, and these hits were further characterized by size-exclusion chromatography. The sequence alignment of monomeric clones showed that all N2C binders were derivatives of one clone (DARPins D1–D5, with 1–4 amino-acid differences between them), but the N3C binders fell into two different subpopulations (DARPins D6 and D8, with one amino-acid difference between them, and DARPin D7) (Supplementary Fig. S1). Finally, the binding kinetics of eight selected DARPins were recorded by surface plasmon resonance. All binders revealed dissociation constants (*K*_d_) of between 4.5 n*M* (DARPin D6) and 8.1 n*M* (DARPin D3) (Supplementary Table S2).

### 3.2. Structure determination of the HER3d4–DARPin D5 complex   

Two data sets for the HER3d4–DARPin D5 complex (N2C binder) were collected from crystals that were obtained under very similar conditions (Table 2 ▸). The data processing suggested two different indexing solutions: either space group *P*2_1_, with unit-cell parmeters *a* = 64.87, *b* = 62.25, *c* = 74.54 Å, β = 102.41°, or space group *P*2_1_2_1_2_1_, with unit-cell parmeters *a* = 62.26, *b* = 64.95, *c* = 144.90 Å. In the monoclinic setting the structure comprises two complexes that are related by a twofold noncrystallographic symmetry (NCS) axis running almost parallel to the crystallographic *c* axis (0.13° tilt between the NCS and unit-cell axes). In the orthorhombic setting two complexes are related by the translational NCS vector (0.00, 0.76, 0.50). In fact, the diffraction data from the orthorhombic crystals can be processed in the monoclinic space group, albeit with significantly impaired merging statistics (*R*_meas_ and *R*_p.i.m._ of 14.5% and 5.9%, respectively, in the monoclinic setting, compared with 7.8% and 2.3% in the orthorhombic setting for low-resolution data up to approximately 6 Å).

**Figure 1:**
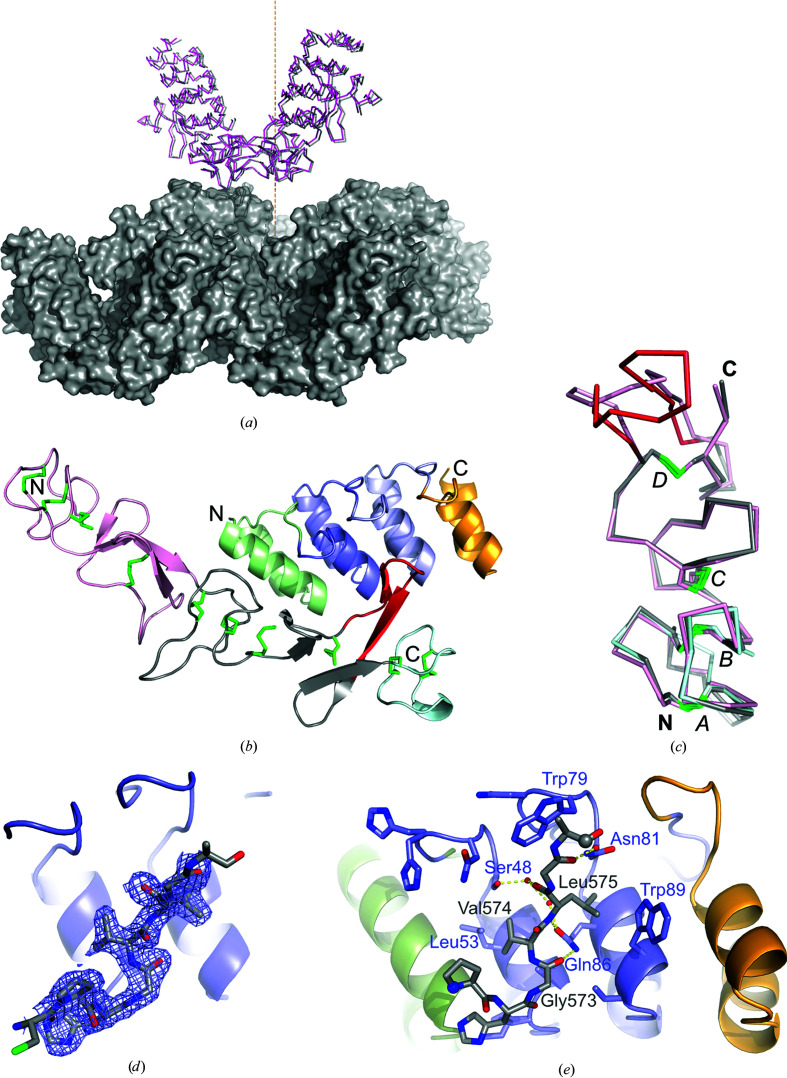
Details of the HER3d4–DARPin D5 complex. (*a*) Superposition of the HER3d4–DARPin D5 complexes in the orthorhombic (gray) and monoclinic (magenta) settings. Molecules within the same *ab* layer are shown as molecular surfaces in gray. The unit-cell *c* axis in the orthorhombic setting is shown as an orange dotted line. (*b*) Overview of the complex. The DARPin D5 N-cap, internal repeats 1 and 2 and the C-cap are shown as cartoons in green, dark blue, light blue and orange, respectively. HER3d4 furin-like domains 1–3 are colored pink, gray and cyan. Cysteines are shown as green sticks. The protruding loop is highlighted in red (residues 571–584). (*c*) Superposition of the furin-like cysteine-rich domains of HER3d4. Coloring is as in (*b*). The N- and C-termini are labeled in bold. Disulfide bridges (*A*–*D*) are labeled in italics. (*d*) The 2*mF*_o_ − *DF*_c_ electron-density map for HER3d4 residues 571–576 was contoured at 1.2σ. (*e*) Details of the DARPin D5–HER3d4 interface. HER3 residues are shown with gray C atoms and chain breaks are highlighted by spheres. Residues at randomized DARPin positions and the framework residues Leu53 and Gln86 are shown as blue sticks, hydrogen bonds as yellow dashed lines and water molecules as red spheres.

Both crystal lattices are constructed by molecular layers of tightly connected HER3d4–DARPin D5 complexes within the *ab* plane (sum of crystal contact areas 3136 Å^2^). Perpendicular to the *ab* plane these layers are only weakly connected (crystal contact area 173 Å^2^). A comparison of the lattices revealed that the packings are almost identical. The only obvious difference is a small shift between the *ab* planes relative to each other of less than 1 Å (Fig. 1 ▸ *a*). The *ab* planes are connected by a crystal contact involving HER3d4 residues 512–513 and residues 541*–542* from the next layer. Therefore, the two crystal forms are almost isomorphic with regard to the crystal packing.

### 3.3. Structure of the complex   

The similarity of the crystal lattices suggests that the HER3d4–DARPin D5 complex structures are also very similar. Indeed, superposition of NCS-related chains within each of the crystal forms *P*2_1_ and *P*2_1_2_1_2_1_ revealed root-mean-square deviations (r.m.s.d.s) for all atoms of 0.164 Å (1554 atoms) and 0.304 Å (1657 atoms), respectively. For the comparison between crystal forms, the upper and lower r.m.s.d.s for the pairwise comparisons are 0.168 and 0.236 Å, respectively. Fig. 1 ▸(*b*) gives an overview of the HER3d4–DARPin D5 complex. As has been pointed out before, the structure of HER3d4 is sparse in secondary-structural elements and lacks a defined hydrophobic core (Cho & Leahy, 2002 ▸). Instead, the structure is stabilized by ten disulfide bridges (Cys481–Cys490, Cys485–Cys498, Cys501–Cys510, Cys514–Csy530, Cys533–Cys546, Cys537–Cys554, Cys557–Cys566, Cys570–Cys591, Cys594–Cys602 and Cys598–Cys610). These disulfides are arranged in an (*ABABCCDD*)_2_ *ABAB* pattern, with the same letters forming one disulfide bond. This suggests that HER3 domain 4 can be further subdivided into three furin-like cysteine-rich subdomains (Wang *et al.*, 2013 ▸) comprising residues 481–532 (subdomain A), 533–593 (subdomain B) and 594–611 (subdomain C). The subdomains are again structurally similar among each other: the r.m.s.d.s for the superposition of subdomains B and C on subdomain A are 0.662 Å (134 atoms) and 0.387 Å (76 atoms), respectively (Fig. 1 ▸ *c*). Subdomains A and B contain eight cysteine residues that are connected by seven loops. Although the sequences are very diverse, all loops except loops 2 and 7 have the same length. Loops 2 and 7 are longer by four and five residues in subdomain B than in subdomain A, respectively. Subdomain C lacks four cysteine residues at the C-terminus, but nevertheless we find the same pattern of loop lengths for the first three loops. This disulfide pattern is conserved in the other HER family members; however, the lengths of loops 2 and 7 in subdomain B differ slightly. HER4 possesses a single amino-acid insertion in loop 2. In loop 7 we find one- and two-amino-acid insertions in HER1 and HER2, respectively, compared with HER3 and HER4, which have the same length. Since the N-terminal strand of loop 7 in subdomain B is recognized by DARPin D5, the conformation and sequence of this loop is probably crucial for the selectivity of DARPin D5 for HER3 (Supplementary Fig. S2).

The binding between HER3d4 and DARPin D5 buries a molecular surface area of between 803 and 823 Å^2^ (Table 5 ▸). DARPin D5 recognizes subdomain B of HER3d4, and predominantly loops 1 and 7. The N-terminal strand of loop 7 (residues 568–577) rests against the second internal D5 repeat, whereas the space between the first internal D5 repeat and HER3d4 is filled by a network of well defined water molecules (Figs. 1 ▸ *d* and 1 ▸ *e*). The molecular surfaces of D5 and HER3d4 do not seem to fit exceptionally well, which is illustrated by the poor surface complementarity indices of between 0.66 and 0.72 (Table 5 ▸), leaving space for water-mediated interactions across the interface, which is consistent with relatively fast on-rates. Loop 1 from HER3d4 subdomain B (residues 532–536) interacts with the DARPin D5 N-cap. Almost all residues at the randomized positions of DARPin D5 become buried upon binding HER3d4, but none of them form direct hydrogen bonds to the target (Fig. 1 ▸ *e*). Instead, residues 13, 16 and 20 from the N-cap, residue 111 from the C-cap and the framework mutation L86Q from the second internal repeat of DARPin D5 form specific hydrogen bonds to HER3d4. Most hydrogen-bond partners on HER3d4 are main-chain atoms. The only side-chain atoms involved in hydrogen bonds are the O^γ^ atoms from Ser532 and Ser568 at the periphery of the interface (Table 5 ▸).

The binding interface involves several hydrophobic interactions, particularly for the recognition of the side chains of Val574 and Leu575, as well as the C^α^ atom of Gly576 from HER3d4 loop 7, which is at van der Waals distance from the bulky side chain of Trp79 of DARPin D5. The side chain of Leu575 is completely shielded from solvent and packs into a hydrophobic pocket formed by the atoms Asn8 CB, Gln86 CA and Trp89 CD1 of DARPin D5. In contrast to this, Val574 packs between the Ser48, Leu53 and Ala56 side chains, but is also contacted by several water molecules (Fig. 1 ▸ *e*). The recognition is dominated by the side chains at internal repeat positions 6, 10 and 13. Positions 6 and 13 are randomized in the library, but position 10 belongs to the DARPin framework. Position 10 is occupied by leucine in the first internal repeat (residue Leu53), but in the second internal repeat this leucine is mutated to glutamine, a consequence of the mutagenesis inherent in ribosome display. The L86Q framework mutation positions the hydrophilic Gln86 side chain between the Val574 and Leu575 side chains, where it forms hydrogen bonds to the HER3d4 main-chain atoms Gly573 O and Leu575 N, thus explaining its selection.

### 3.4. Comparison to other structures   

The extracellular domains (ECDs) of members of the HER family can adopt at least two different conformations. In the ligand-free state, the ECD adopts a tethered conformation in which two protruding loops, one from domain 2 (residues 243–255) and one from domain 4 (residues 571–585), interact and thus lock the ligand-binding domains 1 and 3 in a closed conformation. In this tethered conformation, HER family members are monomers. Upon ligand binding, the intra­molecular interactions between these loops are broken and the ECD stands upright to form a dimer with the ECD from another receptor. In this extended conformation, the protruding loops from domains 2 and 4 participate in formation of the ECD dimer interface (Cho & Leahy, 2002 ▸).

**Figure 2:**
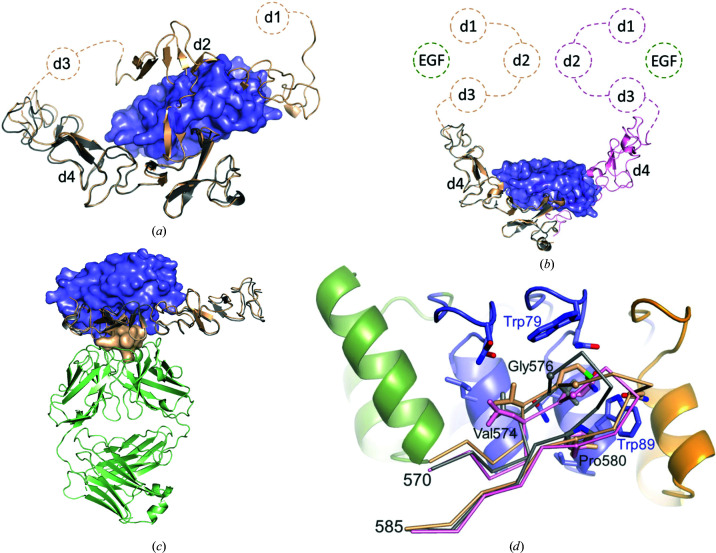
Comparison of the HER3d4–DARPin D5 complex with other structures. DARPin D5 is shown as a transparent blue surface and HER3d4 as a gray cartoon. (*a*) Superposition on the full-length HER3 ECD (PDB entry 1m6b) shown as a cartoon in wheat. To improve clarity, domains 1 and 3 are sketched. Domains are labeled d1–d4. (*b*) Superposition on the extended HER1–EGF complex (PDB entry 3njp), shown as wheat and light pink cartoons. The HER1 domains d1–d3 and EGF are sketched as dashed circles. (*c*) Superposition on the HER3–MOR09825 complex (PDB entry 4p59). The Fab fragment MOR09825 is shown as a light green cartoon and the cognate HER3 is in wheat, where the protruding loop from domain 2 and the entire domain 3 are depicted as a surface and a cartoon, respectively. (*d*) Superposition of HER3d4–DARPin D5 colored as in Fig. 1 ▸(*e*) on the protruding loop 7 from HER1 (PDB entry 3njp, wheat) and HER4 (PDB entry 3u9u, light pink). Amino-acid numbering refers to HER3d4–DARPin D5. The superposition is based on all domain 4 residues, but only loop 7 is depicted for the sake of clarity.

Domain 4 of the HER family is rather rigid, as demonstrated by the similarity among the corresponding domains of the different family members (Cho *et al.*, 2003 ▸). While no structure has been reported for the extended conformation of HER3, both tethered and extended states have been resolved for EGFR (HER1) and HER4 (Liu *et al.*, 2012 ▸; Bouyain *et al.*, 2005 ▸; Li *et al.*, 2005 ▸, 2008 ▸; Matsuda *et al.*, 2018 ▸; Lu *et al.*, 2010 ▸). In these cases, the differences within domain 4 are rather small, underlining the rigidity of this domain. It is thus not surprising that HER3d4 in complex with DARPin D5 adopts a similar conformation to that in the tethered state of the full-length HER3 ECD (PDB entry 1m6b; Cho & Leahy, 2002 ▸), with an r.m.s.d. of 0.932 Å (771 atoms) (Fig. 2 ▸ *a*). The structure is also similar to the structure of HER1 domain 4 in the extended conformation, as an example of an extended structure (PDB entry 3njp; Lu *et al.*, 2010 ▸), with an r.m.s.d. of 0.817 Å (660 atoms) (Fig. 2 ▸ *b*).

Thus, HER3d4 acts as a rigid body and the binding of DARPin D5 does not perturb its overall conformation. The superposition reveals that DARPin D5 exactly recognizes the protruding loop 7 from domain 4 subdomain B that forms the tether. The HER3d4 residue Leu575 seems to be crucial for both interactions, because in the tethered full-length ECD structure Leu575 O forms a hydrogen bond to Tyr246 N from the protruding loop of domain 2 (2.80 Å), and in the DARPin D5 complex Leu575 N forms a hydrogen bond to Gln86 OE1 (Table 5 ▸). Thus, DARPin D5 could prevent the tethered conformation of the HER3 ECD, because DARPin D5 occupies a position which is similar to HER3 domain 2 (Fig. 2 ▸ *a*), but it probably also interferes with interactions of the activated state (see below).

In the extended conformation of the HER1 (EGFR) ECD, the protruding loops of the HER1 domains 4 are only weakly associated. The superposition shows that Ala573 O is at van der Waals distance from Leu582 CD2. In the HER3d4–DARPin D5 complex, the equivalent His572 O is also at van der Waals distance from Ala56 O of DARPin D5 (Fig. 2 ▸ *b*).

Due to the implication of HER3 in various cancers, several structures of the HER3 ECD in complex with antibody Fab fragments, such as 3379, RG7116, MOR09825 and MF3178, are available (PDB entries 5cus, 4leo, 4p59 and 5o4o; Mirschberger *et al.*, 2013 ▸; Lee *et al.*, 2015 ▸; Garner *et al.*, 2013 ▸; Geuijen *et al.*, 2018 ▸). However, only the Fab fragment MOR09825 binds to domain 4, whereas the other Fab fragments target the ligand-binding domains 1 and 3. The comparison shows that DARPin D5 and MOR09825 (Garner *et al.*, 2013 ▸) recognize the protruding HER3d4 loop from opposite directions, such that the epitopes are significantly different from one another (Fig. 2 ▸ *c*). Furthermore, the epitope for MOR09825 involves residues from HER3 domains 2 and 4, whereas the binding of DARPin D5 is independent of domain 2. Therefore, it can be expected that MOR09825 stabilizes the tethered state, whereas DARPin D5 probably interferes with it. However, DARPin D5 most likely additionally interferes with the formation of an activated HER3 heterodimer, and thus with the formation of an oncogenic unit. The ability of MOR09825 to prevent the ligand-dependent and ligand-independent activation of HER3 has previously been shown in cellular assays and *in vivo* models (Garner *et al.*, 2013 ▸).

## 4. Discussion   

Receptor tyrosine kinases from the HER family are attractive targets for the design of anticancer compounds, and the successful development of therapeutic antibodies, such as the HER2-directed trastuzumab and pertuzumab, reveal that targeting the ECD is a viable strategy to identify potent drugs. Using ribosome display in combination with a DARPin library, we identified and characterized eight HER3-directed DARPins that can be grouped into three different families. Family 1 contains the N2C DARPins D1–D5 with single amino-acid substitutions between them at positions 32, 35, 45, 93 and 102. Families 2 (D6 and D8) and 3 (D7) contain DARPins with three internal repeats (Supplementary Fig. S1). All DARPins bind the HER3d4 target with high affinity (Supplementary Table S2). We focused here on the crystallization of the HER3d4–DARPin D5 complex, because of the multiple appearance of similar sequences in the selection and its clear specificity for HER3 (Supplementary Fig. S2).

The high-resolution crystal structures of HER3d4 in complex with DARPin D5 revealed that the DARPin L86Q framework mutation obtained during ribosome display, which is present in DARPins D1–D5, positions the hydrophilic Gln86 side chain in a virtually hydrophobic environment (Fig. 1 ▸ *e*). Normally, placing a hydrophilic side chain into a hydrophobic environment would be energetically unfavorable, but here the Gln86 side chain forms two hydrogen bonds to the HER3d4 main-chain atoms Gly573 O and Leu575 N, which inverts the energetic contribution of this mutation to make it favorable and thus explains its selection in ribosome display.

Residues 571–583 from the second furin-like cysteine-rich domain (subdomain B) of HER3d4 fold into a short β-hairpin. This loop not only forms the epitope for DARPin D5, but is also crucial for the HER3 ECD to adopt its two prevailing conformations, which are the tethered conformation of the monomeric signaling-inactive state in the absence of the HER3 ligand and the extended conformation after ligand binding and subsequent dimerization. In the tethered conformation, residues 571–583 interact with the HER3 domain 2 (Fig. 2 ▸ *a*). There is no reported structure of extended HER3, and in the extended conformation of the homologous HER1 the equivalent residues participate in the formation of the HER1 dimer interface. Assuming that the topology of the activated HER3 receptor is structurally similar to that of its HER1 homologue, DARPin D5 could prevent the formation of an activated HER3 heterodimer, because DARPin D5 occupies the position of the second protomer (Fig. 2 ▸ *b*).

Despite the similarity of domain 4 between the different members of the HER family, DARPin D5 is specific for HER3, and we wanted to understand this selectivity from a structural point of view. Fig. 2 ▸(*d*) shows a superposition of subdomain B loop 7 from HER3d4–DARPin D5 on HER1 (Lu *et al.*, 2010 ▸) and HER4 (Hollmén *et al.*, 2012 ▸). Loop 7 from HER2 has not been included in this comparison because this loop is generally disordered and not resolved in the structure (for example in PDB entries 1s78, 5my6, 6bgt and 1n8z). Since DARPin D5 mainly forms hydrogen bonds to HER3d4 main-chain atoms, it could bind other HER family members as well. Glycine at position 576, which is in van der Waals contact with Trp79, is also conserved in HER1 and HER4. However, Val574 and Leu575, which point towards the hydrophobic interface area of DARPin D5, are conserved in HER1 but are replaced by Leu572 and Gln573 in HER4. HER1 contains a single amino-acid insertion in loop 7, causing a different conformation of HER1 residues 580 and 581 and ultimately a clash between Asn580 and Trp89 from DARPin D5 in this superposition (Fig. 2 ▸ *d*). The side chain of Trp89 from DARPin D5 would also clash with bulky side chains at position 580, which is leucine in HER1 and phenylalanine in HER4, in contrast to proline in HER2 and HER3. In summary, the sequence differences of HER1 and HER4 in loop 7 are most likely to prevent the recognition of those HER family members by DARPin D5.

## 5. Related literature   

The following reference is cited in the supporting information for this article: Kramer *et al.* (2010 ▸).

## Supplementary Material

PDB reference: DARPin D5–HER3 domain 4 complex, orthorhombic crystals, 7bhf

PDB reference: monoclinic crystals, 7bhe

Supplementary Figures and Tables. DOI: 10.1107/S2053230X21006002/no5185sup1.pdf

## Figures and Tables

**Table 1:** Macromolecule-production information

	DARPin D5	HER3 domain 4
Source organism	Artificial	*Homo sapiens*
DNA source	Synthetic	cDNA
Forward primer	AGAGGATCGCATCACCATCACCATCACGGATCCGACCTGGG	TACATTTCTTACATCTATGCACATCACCATCACCATCACTGTGACCCACTGTGCTCC
Reverse primer	ATCTGCTTCGGCCTTCGCTTTAGCATCTGCCGCCGCTTTCG	TTACCAATACTTAAGCTATCATGTCAGATGGGTTTTGCC
Cloning vector	pRDVLDnew_ΔmCherry	pFL
Expression vector	pQiq	EMBacY
Expression host	*E. coli* XL1 Blue	Sf9
Complete amino-acid sequence of the construct produced†	MRGSHHHHHHGGGGSLEVLFQ|GPGSDLGKKLLEAARAGQDDEVRILMANGADVNAFDHNGSTPLHLAAAIGHLEIVEVLLKYGADVNAEDNWGNTPLHQAAWVGHLEIVEVLLKNGADVNAQDKFGKTAFDISIDNGNEDLAEILQKLN	HHHHHHCDPLCSSGGCWGPGPGQCLSCRNYSRGGVCVTHCNFLNGEPREFAHEAECFSCHPECQPMEGTATCNGSGSDTCAQCAHFRDGPHCVSSCPHGVLGAKGPIYKYPDVQNECRPCHENCTQGCKGPELQDCLGQTLVLIGKTHLT

† The vertical line shows the cleavage site for HRV 3C protease.

**Table 2:** Crystallization

Crystal form	Monoclinic	Orthorhombic
Method	Vapor diffusion, sitting drop	Vapor diffusion, sitting drop
Plate type	2 Drop MRC-UVXPRO	2 Drop MRC-UVXPRO
Temperature (K)	277	277
Protein concentration (mg ml^−1^)	7.2	7.2
Buffer composition of protein solution	10 m*M* sodium phosphate, 140 m*M* NaCl pH 7.4	10 m*M* sodium phosphate, 140 m*M* NaCl pH 7.4
Composition of reservoir solution	0.1 *M* sodium citrate, 12% PEG 4000, 0.2 *M* lithium sulfate pH 4.83	0.1 *M* sodium citrate, 10% PEG 4000, 0.2 *M* lithium sulfate pH 4.74
Volume and ratio of drop	200 nl, 1:1	200 nl, 1:1
Volume of reservoir (µl)	75	75

**Table 3:** Data collection and processing Values in parentheses are for the outer shell.

PDB code	7bhe	7bhf
Diffraction source	SLS beamline X06SA	SLS beamline X06SA
Wavelength (Å)	1.000043	1.000043
Temperature (K)	100	100
Detector	EIGER X 16M	EIGER X 16M
Crystal-to-detector distance (mm)	164.960	165.017
Rotation range per image (°)	0.1	0.1
Total rotation range (°)	360	360
Exposure time per image (s)	0.05	0.05
Space group	*P*2_1_	*P*2_1_2_1_2_1_
*a*, *b*, *c* (Å)	64.87, 62.25, 74.54	62.26, 64.95, 144.90
α, β, γ (°)	90, 102.41, 90	90, 90, 90
Mosaicity (°)	0.40	0.20
Resolution range (Å)	44.41–2.30 (2.34–2.30)	38.19–2.00 (2.03–2.00)
Total No. of reflections	110108 (5555)	342098 (15606)
No. of unique reflections	24338 (1250)	38572 (1904)
Completeness (%)	93.2 (97.4)	94.7 (96.5)
Multiplicity	4.52 (4.44)	8.87 (8.20)
〈*I*/σ(*I*)〉	5.4 (2.1)	7.4 (1.4)
*R*_meas_	0.147 (0.576)	0.230 (1.780)
CC_1/2_	0.993 (0.840)	0.991 (0.387)
Overall *B* factor from Wilson plot (Å^2^)	26	21

**Table 4:** Structure refinement Values in parentheses are for the outer shell.

PDB code	7bhe	7bhf
Resolution range (Å)	40.74–2.30 (2.38–2.30)	38.16–2.00 (2.07–2.00)
Completeness (%)	93.14 (97.22)	94.41 (95.50)
σ Cutoff	*F* > 0.000σ(*F*)	*F* > 0.000σ(*F*)
No. of reflections, working set	23143 (2391)	36594 (3633)
No. of reflections, test set	1184 (126)	1834 (184)
Final *R*_work_	0.1766 (0.2198)	0.2094 (0.3280)
Final *R*_free_	0.2450 (0.2876)	0.2636 (0.3773)
Cruickshank DPI	0.337	0.191
No. of non-H atoms		
Protein	3826	3892
Ligand	30 [GOL, ACT]	8 [ACT]
Solvent	463	690
Total	4319	4590
R.m.s. deviations		
Bonds (Å)	0.012	0.012
Angles (°)	1.59	1.54
Average *B* factors		
Overall	34.4	31.3
Protein	33.8	29.6
Ligand	54.1 [GOL, ACT]	39.8 [ACT]
Solvent	48.3	40.8
Clashscore	3.51	3.74
Ramachandran plot		
Most favored (%)	97.63	97.23
Allowed (%)	2.37	2.77
Outliers (%)	0.00	0.00

**Table 5:** Hydrogen bonds, surface area and surface complementarity at the HER3d4–DARPin D5 interface Only distances less than 3.6 Å are given.

		Distance in *P*2_1_ crystals (Å)		Distance in *P*2_1_2_1_2_1_ crystals (Å)	
Atom in DARPin D5	Atom in HER3d4	Chains *A*/*B*	Chains *C*/*D*	Chains *A*/*B*	Chains *C*/*D*
Asp13 OD2	Ser532 OG	3.12	3.29	2.85	3.07
Lys16 NZ	Ser549 O	3.22	3.55		
Glu20 O	Ser568 OG	2.67	2.80	2.69	2.74
Glu20 OE2	Ser568 N	2.95	2.95	3.09	2.87
Glu20 OE2	Ser568 OG	2.67	3.06	3.41	3.28
Gln86 NE2	Gly573 O	2.74	2.89	2.99	2.90
Gln86 OE1	Leu575 N	2.86	3.00	2.87	2.94
Lys111 NZ	Ala577 O	2.73	2.78	2.75	2.77
Surface area† (Å^2^)		803	811	823	810
Surface complementarity		0.72	0.69	0.67	0.66

† Total area buried in the interface as defined by *SC* (Lawrence & Colman, 1993 ▸).
