# Supplementary material for: Crystal structures of HER3 extracellular domain 4 in complex with the designed ankyrin-repeat protein D5
Source: Acta Crystallogr F Struct Biol Commun. 2021 Jun 29;77(Pt 7):192–201. doi: 10.1107/S2053230X21006002 (PMC8248824; doi:10.1107/S2053230X21006002)
Supplement: Supplementary file 1 [file f-77-00192-sup1.pdf]

Acta Cryst F

STRUCTURAL BIOLOGY
COMMUNICATIONS

Volume 77 (2021)

Supporting information for article:

Crystal structures of the HER3 extracellular domain 4 in complex with the designed ankyrin-repeat protein D5

Filip Radom, Clemens Vonrhein, Peer R. E. Mittl and Andreas Plückthun

```
           |           N-cap                  |           1st repeat           |
                    20        30        40        50        60        70        80
           ....|....|....|....|....|....|....|....|....|....|....|....|....|....|
consensus  GSDLGKKLLEAARAGQDDEVRILMANGADVNAxDxxGxTPLHLAAxxGHLEIVEVLLKzGADVNAxDxxG
D1         ........................T.......F.HN.S.......AI...........Y......E.NW.
D2         .....................V..........F.HN.S.......AI...........Y......E.NW.
D3         ................................F.HS.S.......AI...........Y......E.NW.
D4         ................................F.HN.S.......AI...........Y......E.NW.
D5         ................................F.HN.S.......AI...........Y......E.NW.
D6         ................................Q.VS.Q.......IW...........T......W.HW.
D8         ................................Q.VS.Q.......IW...........T......W.HW.
D7         ...........T....................Q.VS.Q.......IW...........T......Q.WF.

                 2nd repeat           |          3rd repeat            |
                    90        100       110       120       130       140       150
           ....|....|....|....|....|....|....|....|....|....|....|....|....|....|
consensus  xTPLHLAAxxGHLEIVEVLLKzGTDVNAxDxxGxTPLHLAAxxGHLEIVEVLLKzGADVNAQDKFGKTPF
D1         N....Q..WV..R........Y.V....---------------------------------Q......A.
D2         N....Q..WV..R........Y.A....---------------------------------Q......A.
D3         N....Q..WV..R........Y.A....---------------------------------Q......A.
D4         N....Q..WV..R........Y.A....---------------------------------Q......A.
D5         N....Q..WV...........N.A....---------------------------------Q......A.
D6         N.......WQ...........A......K.SE.F.......WW...........H......Q........
D8         N.......WQ...........A.A....K.SE.F.......WW...........H......Q........
D7         E.......VQ...........T.A....W.AY.H.......NY...........H......Q........

             C-cap
                    160       170
           ....|....|....|....|..
consensus  DLAIDNGNEDIAEVLQKAAKLN
D1         .IS.......L..I..---...
D2         .IS.......L..I..---...
D3         .IS.......L..I..---...
D4         .IS.......L..I..---...
D5         .IS.......L..I..---...
D6         ......................
D8         ......................
D7         ......................
```

Figure S1 Alignment of HER3d4 binding DARPins D1 to D8. Randomized positions, where any amino acid except cysteine, glycine or proline is allowed, are indicated by “x”. Positions where only asparagine, histidine or tyrosine is allowed are indicated by “z”. Ten residues at the N-terminus comprising the MRGS-His_6 tag are not shown. D1 to D5 are N2C DARPins with two internal repeats, D6 to D8 are N3C DARPins with 3 internal repeats. D1 to D5 contain the first generation C-cap (Binz *et al.*, 2004) and while the N3C DARPins contained the more stable second generation C-cap (Kramer *et al.*, 2010).

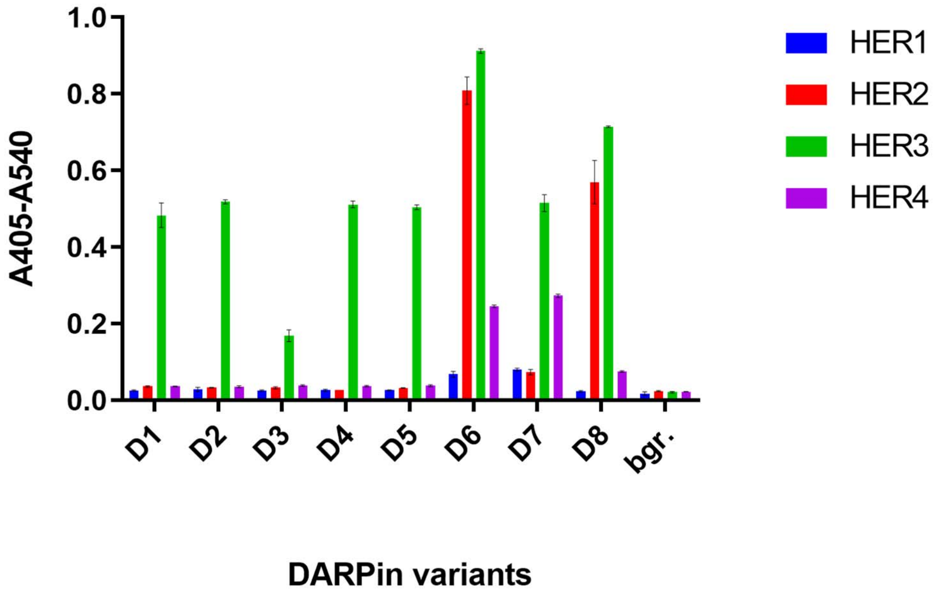

Figure S2 Cross-specificity analysis of HER3 binders with ELISA. Full extracellular domains (ECDs) of HER family members, i.e., HER1 (EGFR), HER2, HER3 and HER4 (SinoBiological 10001-H08H, 10004-H08H 10201-H08H, 10363-H08H) were immobilized on MaxiSorp™ plates (Nunc) at 20 nM concentration by direct coating. DARPins at 50 nM concentration were allowed to bind. Binding was detected with mouse anti-FLAG (Sigma, F3165) antibody at 1:5000 dilution, followed with goat anti-mouse coupled to alkaline phosphatase (Sigma, A3562) antibody at 1:10000 dilution. Each incubation step was for 1 h at 4°C. Binding was detected by turnover of paranitrophenylphosphate (pNPP) at 405 nm and corrected for background signal at 540 nm. *bgr*, background control w/o DARPins. The assay was performed in parallel duplicates and the error bars represent SEM.

Table S1 Ribosome display selection conditions.

Round	Prepanning (pmol)		Panning (pmol)		Compet.[d]	Wash[e]
	Immobil.[a]	Protein[b]	Immobil.[a]	Protein[c]		
1	—	—	N, 24	60	—	5 × 5"
2	S, 20	50	S, 8	20	—	5 × 5'
3	N, 10	25	N, 2	5	—	5', 2 × 6', 7', 6'
error-prone PCR (3 µM dPTP, 3 µM 8-oxo-dGTP)						
4	S, 10	25	S, 0.8	2	100×	2', 5', 2 × 6', 2 × 7', 6'
5	N, 30	75	N, 30	75	—	2', 5', 2 × 6', 2 × 7', 6'

[a] Immobilization with: N — neutravidin, S — streptavidin,

[b] Protein used in prepanning: HER2d4. Prepanning time was 1 h.

[c] Protein used in panning: HER3d4. Panning time was 1 h.

[d] Competition for off-rate selection where indicated. Fold excess of non-biotinylated target. Incubation time was 1 h.

[e] Washing time in minutes (') and seconds (")

Table S2 Dissociation constants of HER3d4:DARPin complexes.

DARPin	Library	k_{on} ($M^{-1}s^{-1}$)	k_{off} (s^{-1})	K_d (nM)	Chi^2
D1	N2C	1.23×10^{6}	9.32×10^{-3}	7.6	6.9
D2	N2C	1.37×10^{6}	7.83×10^{-3}	5.7	12.7
D3	N2C	1.35×10^{6}	1.09×10^{-2}	8.1	4.7
D4	N2C	1.51×10^{6}	6.69×10^{-3}	4.4	20.7
D5	N2C	1.25×10^{6}	8.56×10^{-3}	6.9	9.0
D6	N3C	1.38×10^{5}	6.23×10^{-4}	4.5	3.2
D7	N3C	1.96×10^{5}	1.47×10^{-3}	7.5	3.2
D8	N3C	1.58×10^{5}	7.91×10^{-4}	5.0	3.9
